# The effectiveness of the “Brainwork Intervention” in reducing sick leave for unemployed workers with psychological problems: design of a controlled clinical trial

**DOI:** 10.1186/s12889-015-1728-z

**Published:** 2015-04-14

**Authors:** Selwin S Audhoe, Karen Nieuwenhuijsen, Jan L Hoving, Judith K Sluiter, Monique HW Frings-Dresen

**Affiliations:** Academic Medical Center, Department: Coronel Institute of Occupational Health/ Research Center for Insurance Medicine, University of Amsterdam, PO BOX 22700, 1100 DE Amsterdam, The Netherlands

**Keywords:** Unemployment, Participation, Return-to-work, Sick leave, Vocational rehabilitation, Intervention, Counselling, Psychological problems

## Abstract

**Background:**

Among the working population, unemployed, temporary agency and expired fixed-term contract workers having psychological problems are a particularly vulnerable group, at risk for sickness absence and prolonged work disability. Studies investigating the effectiveness of return-to-work (RTW) interventions on these workers, who are without an employment contract, are scarce. Therefore, a RTW intervention called ‘Brainwork’ was developed. The objective of this paper is to describe the ‘Brainwork Intervention’ and the trial design evaluating its effectiveness in reducing the duration of sick leave compared to usual care.

**Methods/Design:**

The ‘Brainwork Intervention’ is designed to assist unemployed, temporary agency and expired fixed-term contract workers who are sick-listed due to psychological problems, with their return to work. The ‘Brainwork Intervention’ uses an activating approach: in the early stage of sick leave, workers are encouraged to exercise and undertake activities aimed at regaining control and functional recovery while job coaches actively support their search for (temporary) jobs. The content of the intervention is tailored to the severity of the psychological problems and functional impairments, as well as the specific psychosocial problems encountered by the sick-listed worker.

The intervention study is designed as a quasi-randomized controlled clinical trial with a one-year follow-up and is being conducted in the Netherlands. The control group receives care as usual with minimal involvement of occupational health professionals. Outcomes are measured at baseline, and 4, 8 and 12 months after initiation of the program. The primary outcome measure is the duration of sick leave. Secondary outcome measures are: the proportion of subjects who returned to work at 8 and 12 months; the number of days of paid employment during the follow-up period; the degree of worker participation; the level of psychological complaints; and the self-efficacy for return to work. The cost-benefit analysis will be evaluated from an insurer’s perspective.

**Discussion:**

The methodological considerations of the study design are discussed. In this trial we evaluate the effectiveness of an intervention in real occupational health practice, rather than under highly controlled circumstances. The results will be published in 2015.

**Trial registration:**

Trial registration number: NTR4190

Date of registration: September 27^th^ 2013

## Background

### Unemployed and temporary agency workers and psychological problems

Workers without an employment contract, such as unemployed and temporary agency workers and workers with expired fixed-term contracts, are at a higher risk for work disability compared to the general working population, as there is no employer to return to when sick-listed or after sick leave has expired [1-7]. Workers without an employment contract represent a vulnerable group within the working population. They are characterized by a poor mental health status and a low socio-economic position [4-7]. They have less job security and are more often of non-native status, with a greater distance to the labour market, and an increased risk for work disability compared to workers with an employment contract [4,5,7]. In recent years, the number of workers without an employment contract has been growing due, in part, to the worldwide economic crisis [4,5,8-10]. As many unemployed individuals will experience psychological problems [11-14], the impact of sickness absence on public and occupational health programs/systems is important. Psychological problems are currently the leading cause of sickness absence in most high-income countries, accounting for approximately 40% of sick leave [15,16]. In the Netherlands, 40% of the sick leave of workers without an employment contract is due to psychological problems [17]. One of the primary problems these workers encounter is loss of control. In absence of an employer, regaining control by gradually returning to work is not possible. However, there are other ways to gain control and activate these workers, particularly through physical activities and volunteer work.

### The Dutch social security system

In the Netherlands, the Sickness Benefits Act provides assistance for sick-listed workers without an employment contract. The Social Security Agency (SSA) provides a sickness benefit during the first two years of sickness absence. There are no legislative mandates for these workers to be returned to their previous/last job. Therefore, the SSA is also responsible for sickness absence counselling, which is usually conducted by an insurance physician (IP).

### Study rationale

Most return-to-work (RTW) intervention research is aimed at sick-listed, currently employed workers with an employment contract. In contrast, the development of effective RTW interventions for workers without an employment contract is lagging behind [18,19]. RTW interventions need to be developed for this group because the existing RTW interventions for employed workers do not address situations in which there is no workplace to which they can return. Focusing on the ability to work is important, as working even some hours during the RTW process is an important predictor of successful RTW in this group [20]. For this purpose, and to optimize the sickness absence counselling, professionals of the SSA developed the Brainwork Intervention for workers without an employment contract, who are sick-listed due to psychological problems. The Brainwork Intervention uses an activating approach, whereby in the early stage of sick leave, the sick-listed workers are encouraged to exercise and undertake other activities concurrently with their job search.

### Objective

The objective of this paper is to describe the ‘Brainwork Intervention’ and present the design of a controlled clinical trial to study its effectiveness in reducing the duration of sick leave for sick-listed unemployed and temporary agency workers and workers with expired fixed-term contracts who have psychological problems and to compare this intervention to the usual care.

## Methods/Design

This study is being conducted as a two-armed quasi-randomized controlled clinical trial with a follow-up period of one year. To describe the design of the trial, the CONSORT statement, was followed [21,22].

### Intervention

#### Brainwork Intervention

To optimize the sickness absence counselling of sick-listed workers without an employment contract who have psychological problems, occupational health (OH) professionals of one of the front offices of the Dutch SSA developed the Brainwork Intervention. The intervention is optimized by categorizing the sick-listed workers and targeting social-medical interventions to the specific categories of workers. The core elements of the Brainwork Intervention are: (1) personal attention given by the OH professional to the sick-listed worker through face-to-face contact, within five working days after the SSA received the sick report; (2) classification of the worker based on the severity of the psychological problems, the degree of functional impairments or loss of control and estimated recovery time of the sick-listed worker by the IP (see Table 1); (3) early activation of the worker and provide structure to the worker by formulating explicit goals and timetables for recovery, the early reintegration into primary paid work and if necessary, enhancing work experience or carrying out volunteer work, and an exercise program; (4) referral of the worker to additional specific psychological and/or social interventions such as psychological treatment (e.g., dealing with coping problems or eye movement desensitization and reprocessing (EMDR) for persons with impaired trauma counselling) and debt counselling; (5) facilitating a timely internal work process for the SSA by optimizing the collaboration between the OH professionals involved (IP, vocational rehabilitation counsellor, labour expert, nurse practitioner, secretary); (6) intensive vocational counselling by the SSA; (7) counselling by a vocational rehabilitation agency.

**Table 1:** **Brainwork category classification**

**Category 0**	**Category 1**	**Category 2**	**Category 3**
estimated recovery	estimated recovery	estimated recovery	estimated recovery
<2 weeks	<3 months	3-12 months	>12 months or unknown
Very mild problems	Mild psychological problems	Moderate- severe psychological problems include somatisation	Severe psychological problems, clinical admission or day care treatment
	***OR***	***OR***	
	Very mild problems with (severe) psychosocial problems and/or inadequate coping	Mild psychological problems with (severe) psychosocial problems and/or inadequate coping style	
	***OR***		
	Moderate- severe psychological problems with adequate coping		
No functional impairments	Functional impairments	Severe functional impairments	Severe functional impairments to inability for functioning
	(loss of control)		

The Brainwork Intervention is applicable for category 1 and 2.

**Figure 1:**
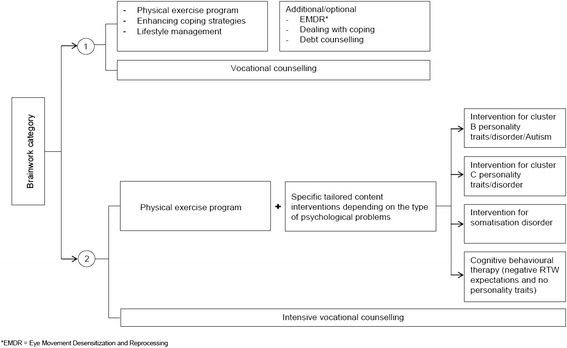
Brainwork Intervention.

The Brainwork Intervention uses an activating approach: in the early stage of sick leave, the sick-listed workers are encouraged to exercise and undertake activities aimed at regaining control and functional recovery, while job coaches actively support their search for (temporary) jobs. The tailored content of the intervention varies depending on the severity of the psychological problems and specific psychosocial problems the sick-listed worker must address. The components of the intervention include an exercise program, vocational training, gym membership and attention tailored to their mental and/or psychosocial problems. All interventions are combined with guidance from vocational rehabilitation agencies and explicit goals and timetables for recovery (see Figure 1 for an overview of the Brainwork Interventions per category). It is expected that this approach will lead to functional recovery and reduction of sick leave duration of the sick-listed worker.

#### Rationale of the Brainwork Intervention

Hereafter, the intended mechanisms of the Brainwork Intervention components are described. (1) Face to face contact: by having a personal conversation at an early stage, the worker will feel as if his problems are taken seriously. This contact encourages the worker to make the required commitment to the intervention, which is required for achieving a positive outcome. (2) Category classification: shortly after inclusion of the worker in the program, the IP classifies the worker into a category based on the IP’s assessment of the worker’s functional impairments and estimated recovery time. A stepped care approach is used, which allows a worker with an estimated favourable recovery (estimated recovery within 3 months) to receive less intense and shorter sickness absence counselling. The classification delineates other elements of the Brainwork Intervention, namely goal-setting, increasing the efficiency of SSA work processes and worker referrals to specific interventions. The IP is asked to provide a recovery estimation of the worker based on the severity of his psychological problems and functional impairments, as well as RTW prognostic factors such as health expectations, RTW expectations and the perceived health of the worker, age, and personal factors such as education level [3,20,23]. (3) Motivate to activate: this element is designed to provide active day care for the worker. In groups with varying psychological disorders, it has been demonstrated that activation promotes recovery from mental complaints and increased functioning [24,25]. (4) Goal-setting: setting explicit goals regarding activity level of the worker and the final RTW date (or maximum duration of the sickness benefit period) is part of the Brainwork Intervention. Defining the expected recovery period will give the Brainwork participant a better perspective on his recovery, increase his sense of control, encourage a faster recovery and reduce the number of psychological complaints [26-28]. (5) Providing advice for daily structure: Brainwork participants are advised to maintain a good day structure by getting up at a specific predetermined time and planning an activity for the morning. The activity can be, for example, a visit to the gym but is not limited to exercise. This advice creates a better day-structure for the worker and may promote the recovery from mental complaints and increased functioning [24,25]. (6) Guidance to work: because work is a structuring activity, it is considered to have a positive impact on the recovery from mental complaints and increased functioning because it leads to a clear day-structure and more active day care. In addition, having a work perspective focuses the worker on improving, which has a positive effect on recovery from complaints and improved functioning. Furthermore, because there is a fast start of activities aimed at RTW and paid labour, a shorter duration of sick leave is anticipated. (7) Increased efficiency of the internal SSA work process: by optimizing the collaboration between multiple OH professionals, the prompt handling of requests regarding sickness benefit claims and quick referral to external intervention partners reduces the turnaround time and facilitates early RTW. (8) Timely referral for interventions: depending on the initial category classification of the worker, a protocol-based quick referral to external intervention partners may follow for those workers for whom a quick recovery is not expected. This method promotes a quick start of the Brainwork Intervention and is considered to have a positive effect on the mental complaints and functional recovery of the worker.

### Usual care

The control group is receiving counselling according to care as usual. This care consisted of minimal involvement by the IP and, to a lesser extent, of other OH professionals. In this scenario, the active sickness absence counselling starts at a later time point during the sick leave process. Furthermore, there is minimal referral to external intervention partners and, if referred, it does not occur using the stepped care approach, which is based on the classification category assigned in the intervention group. Finally, early reintegration into primary paid work or enhancing work experience is not the main goal of usual care. We will record the interventions received by the workers in the control group.

### Study design

**Figure 2:**
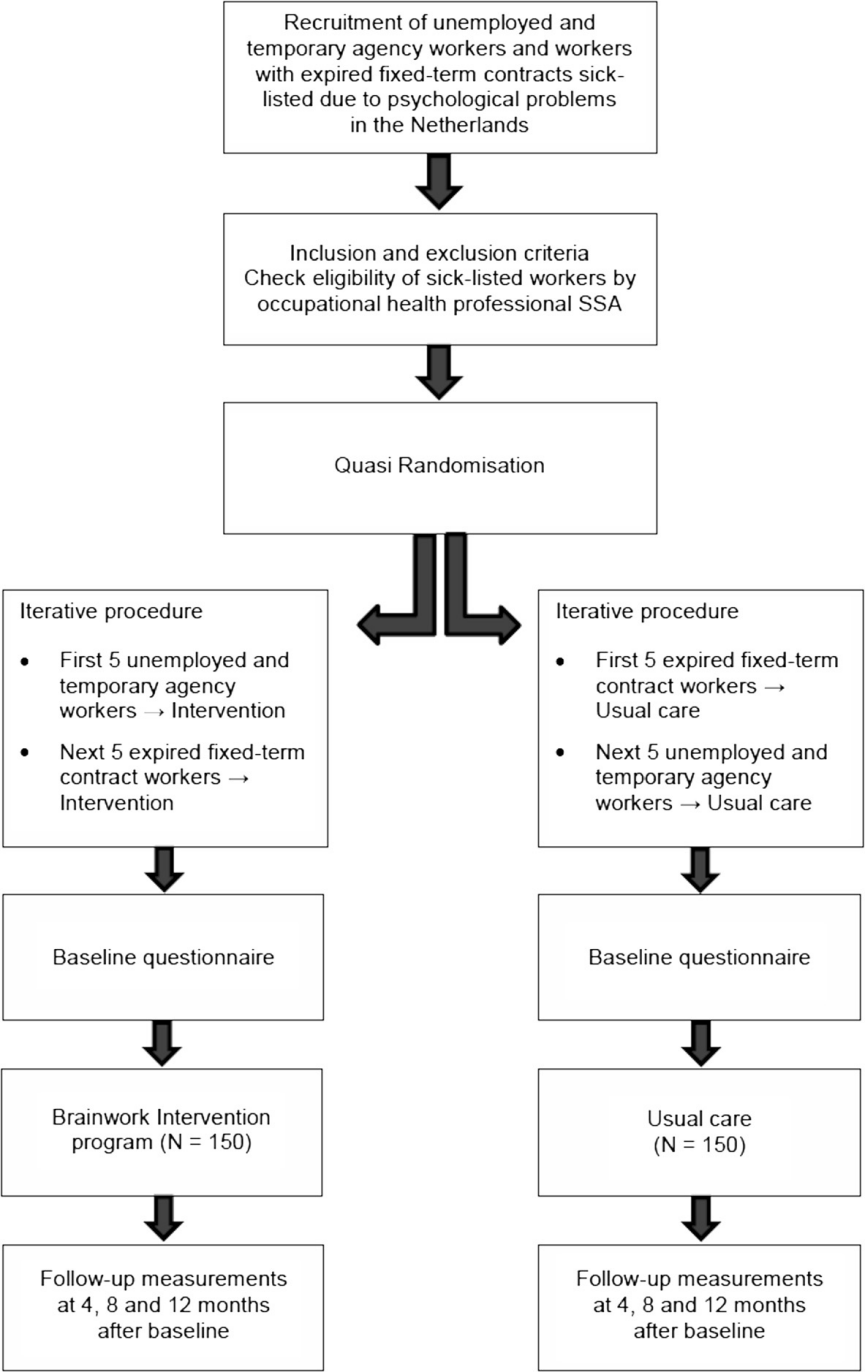
Design of the study.

Participants are allocated to two groups: an intervention group, in which the participants receive the Brainwork Intervention and a control group, in which participants receive usual care (see Figure 2). The study was presented to the Medical Ethics Committee of the Academic Medical Center (AMC), University of Amsterdam. The Medical Ethics Committee declared that the study design did not require comprehensive ethical review, as the Medical Research Involving Human Subjects Act does not apply to this study [29]. Besides the decision of the Medical Ethics Committee, the study has to adhere to Research Code of AMC. This study is listed in the Netherlands Trial Register (NTR) under NTR4190.

### Setting

The controlled clinical trial is conducted in collaboration with three front offices of the Dutch SSA across the Netherlands (Hengelo (east), Rotterdam (south-west), Den Bosch (south)), vocational rehabilitation agencies, mental health institutions/professionals and companies that specialised in activating rehabilitation programmes consisting of physical exercise, dealing with coping and lifestyle management.

### Study population

The population in this study consists of unemployed and temporary agency workers and workers with expired fixed-term contracts, who live in the eastern, south-western or southern part of the Netherlands and when sick-listed are the responsibility of the three participating front offices of the Dutch SSA. The inclusion criteria are: (1) being an unemployed or temporary agency worker or worker with an expired fixed-term contract; (2) between 18 and 64 years of age; (3) sick-listed and not expected to RTW within two weeks after reporting sick or contact with the vocational rehabilitation counsellor of the SSA; (4) having psychological problems/complaints as the main reason for a sickness benefit claim; and (5) adequate command of the Dutch language. The exclusion criteria are: (1) recent pregnancy or up to three months after delivery; (2) substance addiction (alcohol, drugs and medicines) as the main reason for a sickness benefit claim; (3) having a severe psychiatric disorder with an expected recovery of more than one year (e.g., hospitalization or day treatment).

### Procedure

#### Recruitment of participants

The sick-listed workers with psychological problems from one of the three participating front offices of the SSA are included in the study if they meet the inclusion criteria. After inclusion in the study, the workers receive a letter from the staff IP of the appropriate SSA front office, on behalf of the investigators. The purpose of this letter is to provide information about the study and to ask for the workers cooperation (informed consent) in completing questionnaires during the study. In addition, the workers also receive an informational flyer containing additional details about the study, a baseline questionnaire, an informed consent form, a refusal form for those not willing to complete the questionnaires, and a return envelope for the baseline questionnaire or refusal form. At follow-up, only the sick-listed workers who signed an informed consent form to fill the questionnaires are approached. Evaluation of the workers’ questionnaires takes place at baseline, and 4, 8 and 12 months after the SSA received the sick report. The questionnaires are sent to the participants’ home addresses.

#### Recruitment and training of occupational health professionals

At each SSA office, an existing team of OH professionals is designated as an intervention team and one as a control group team. An OH professional team consists of insurance physicians, vocational rehabilitation counsellors, labour experts, at least one nurse practitioner and secretaries. Instruction and coaching sessions are held for all of the OH professionals on the intervention team. Members of this team also receive a syllabus with detailed information about the Brainwork Intervention, the protocol, practical summaries, flowcharts, a schedule of actions and the registration forms to aid in the application of this new intervention. Furthermore, team members receive a two-day training in motivational interviewing. The training provides the OH professionals with the motivational interviewing skills necessary to activate the sick-listed workers’ participation in the Brainwork Intervention, to initiate positive behavioural changes and to address resistance to change of the sick-listed workers.

### Randomisation

Quasi randomisation is conducted at the participant (workers) level at each of the three participating front offices of the Dutch SSA. Because workers with expired fixed-term contracts are registered later during the sick leave process, the Brainwork Intervention also begins later for this group. Therefore, the sick-listed workers are pre-stratified based on the type of worker (i.e., unemployed and temporary versus expired fixed-term contracts) to ensure equal distribution of the different types of workers in the control and intervention group. Because a blinded allocation is impractical and difficult to set up in the practice of the Dutch SSA, the following allocation scheme is used: the first five sick-listed unemployed and temporary agency workers with psychological problems, as identified by each participating Dutch SSA front office, and who met the inclusion criteria are allocated to the intervention group. The first five workers with expired fixed-term contracts having psychological problems are allocated to the control group. The next five sick-listed unemployed and temporary agency workers having psychological problems are allocated to the control group, while the next five workers with expired fixed-term contracts are allocated to the intervention group and so on. The person who allocated the worker to either control or experimental group is unaware of the type or severity of the psychological problem, or of any other participant characteristics.

### Blinding

Participants, OH professionals and intervention partners such as vocational rehabilitation agencies and mental health institutions/professionals are not blinded to the intervention. Blinding was deemed unnecessary because the Brainwork Intervention contains several new elements compared to usual care including, category classification, a protocol-based approach and contracting of vocational rehabilitation agencies. Because the SSA registers sickness benefits, these measurements are derived from the computerised SSA database. Therefore, any bias due to lack of blinding is prevented for the primary outcome. Furthermore, most secondary outcome measures are self-reported, and thus, blinding to the participant groups is not possible. After inclusion in the study, all participants receive a research code. All data will be entered in the computer by a research assistant, using this research code, in order to guarantee that analyses of the data by the researcher will be anonymous.

### Measures

#### Primary outcome

##### Duration of sick leave

The primary outcome measure in this study is duration of sick leave and operationalised as duration of the sickness benefit period (in days) from the first day of reporting sick until the end of the sickness benefit. The sickness benefit ends after a full RTW (e.g., for temporary agency workers) or if the participant is declared fit for work (e.g., for unemployed workers). Data on sickness benefit duration are continuously registered by the SSA and will be acquired from the SSA database six months after inclusion of the last participant and after a one-year follow-up period. Because the SSA registers all the data regarding sickness benefit claims, a loss of data to follow-up with regard to the primary outcome is not expected.

#### Secondary outcomes

##### Proportion of subjects returned to work

The proportion of the subjects who returned to work is operationalised as the proportion who ended sickness benefit claims and will be measured at 8 and 12 months after the date that the SSA received the sick report. Data are acquired from the SSA database.

##### Duration from SSA transfer to RTW

The duration from SSA transfer to RTW is operationalised as the actual duration that the sick-listed worker was under counselling by the front office of the SSA until the end of the sickness benefit. When unemployed and temporary agency workers report themselves sick, first the back office of the SSA is notified. It may take two to four weeks before the front office of the SSA receives the sick report. Only after the front office receives the report can the sickness absence counselling begin. Workers with expired fixed-term contracts (those whose contracts expired while they were sick-listed) will register at the SSA later in the sick leave process than the unemployed and temporary agency workers because the contract workers have an employer at the time of reporting sick. At this registration, the SSA receives all data from the start of the sick leave onwards, from the former employer and occupational health service related to the employer. After expiration of the fixed-term contract, the SSA becomes responsible for the sickness benefit claim, so the actual duration of SSA counselling for this sick-listed worker is shorter then the duration of sick leave. Data are continuously recorded and will be acquired from the SSA database.

##### Number of days of paid employment during follow-up

The number of days of paid employment during the follow-up period will be obtained from both the SSA database and the self-reported information in the questionnaires at 4, 8 and 12 months after the SSA received the sick report.

##### Degree of participation

The degree of participation can vary between being inactive, doing volunteer work, working in a work experience situation (real labour experience without wage) and paid work. The degree of participation will be obtained from both the SSA database and the self-reported information in the questionnaires at 4, 8 and 12 months after the SSA received the sick report (ordinal scale: inactive/volunteer/labour experience/paid work).

##### Psychological complaints

Psychological complaints are measured using the Dutch translation of the General Health Questionnaire-12 (GHQ-12) at baseline, and 4, 8 and 12 months after the SSA received the sick report [30].

##### Self-efficacy for return to work

Self-efficacy as related to work performance after sick leave is measured with a validated RTW self-efficacy questionnaire at baseline, and 4, 8 and 12 months after the SSA received the sick report [31].

##### Costs and benefits from SSA perspective

Costs and benefits from the SSA perspective refer to the incremental costs and benefits of the Brainwork Intervention. Incremental costs are the costs of the Brainwork Intervention minus the costs of the usual care. The incremental benefits are the benefits of the Brainwork Intervention minus the benefits of the usual care. Costs associated with Brainwork are the training and educational costs for the OH professionals, the wage of the OH professionals and the costs of the Brainwork Intervention. Costs for usual care include the wage of the OH professionals and the costs of the interventions if deployed. Benefits for both groups are associated with savings in sickness and unemployment benefit claims. In the context of this study there are only benefits from the SSA perspective if the sick-listed worker returned to paid work after the end of the sickness benefit claim. The ending of the sickness benefit claim by returning to the unemployment benefit is not beneficial for the SSA because the SSA is also responsible for the unemployment benefit claims. Data will be acquired from the SSA database.

An overview of the outcome measures and the measurement instruments used, including a time path for all measurements, is presented in Table 2. Quantitative indicators for process measurement will be obtained from the SSA database.

**Table 2:** **Overview of outcome measures and their instruments and time path**

	**Time path**				
	**Baseline**	**4 months**	**8 months**	**12 months**	**Continuous**
	**T0**	**T1**	**T2**	**T3**	**(until 12 months)**
**Data instruments**					
Dutch SSA* Database					X
Questionnaires 1 to 4	1	2	3	4	
**Measurements**					
**Primary outcome**					
Duration of sick leave					X
*(SSA Database)*					
**Secondary outcome**					
Proportion of subjects returned to work at 8 and 12 months *(SSA Database)*			X	X	
Duration from transfer to the SSA until RTW *(SSA Database)*					X
Number of days of paid employment during follow-up		X	X	X	
*(questionnaire and SSA database)*					
Degree of participation		X	X	X	
*(questionnaire and SSA database)*					
Psychological complaints	X	X	X	X	
*(questionnaire)*					
Self-efficacy for return to work	X	X	X	X	
*(questionnaire)*					
Costs and benefits from SSA perspective					X
*(SSA Database)*					

*Dutch SSA = Dutch Social Security Agency.

### Sample size and power analysis

A power analysis was performed to calculate the required number of participants in this study. The mean and standard deviations of the duration of sick leave of all sick-listed workers without an employment contract having psychological problems who registered at the SSA in 2011 were taken as a starting point. Because there are no data available from previous studies on the expected differences, we only calculated how many participants are needed to display any differences in duration of sick leave. The power analysis using program nQuery Advisor showed that 144 participants are needed per group (288 total) to detect a mean difference in duration of sick leave of 40 days. Therefore, we decided to include 300 participants for this study.

### Data analysis

All statistical analyses will be performed at worker’s level according to the intention-to-treat principle. To check the success of the quasi randomisation procedure similarities in the descriptive statistics will be determined, comparing the baseline measurements of both groups. If necessary, the main analyses will be adjusted for prognostic dissimilarities. For those aspects of the protocol which were fixed for all participants, such as personal attention given by the OH professional to the sick-listed worker through face-to-face contact within five working days after the SSA received the sick report, or timely referral of the worker to the external intervention partner, the protocol deviations will be analyzed. To assess the presence of bias due to protocol deviations, the results of the intention-to-treat analyses will be compared to per-protocol analyses.

#### Effect evaluation

To assess the effectiveness of the Brainwork Intervention for the primary outcome, a Linear Mixed Models (LMM) analysis of the differences between the average sick leave duration (in days) in the intervention and control group will be performed. Therefore, a model with group as fixed factor and the vocational rehabilitation counsellor of the SSA at a primary hierarchical level and the participants at a secondary hierarchical level (where appropriate) will be built. For all participants who have not returned to work after 1 year of follow-up, the value of 365 days of sickness absence will be imputed.

For the secondary outcomes, excepting costs and benefits, LMM analysis for continuous outcomes and Generalized Linear Mixed Models analysis for dichotomous/ordinal outcomes will be performed. Therefore, a model with group as fixed factor and the vocational rehabilitation counsellor of the SSA at a primary hierarchical level and the participants at a secondary hierarchical level (where appropriate) will be built. In the case of differences in the ratios between the types of workers (unemployed and temporary agency versus expired fixed-term contract) due to attrition, a correction for the differences will be performed by including type of worker as a covariate in the analysis.

#### Costs and benefits from SSA perspective

The costs and benefits from SSA perspective will be determined by incremental costs (Brainwork minus control) and incremental benefits (Brainwork minus control) of the Brainwork Intervention. The benefits are savings in sickness and unemployment benefits claims.

## Discussion

In this pragmatic controlled study we will evaluate the effectiveness of an intervention in real occupational health practice. We expect to observe a reduction in sickness absence and an increase in RTW (in paid work) with all potential benefits for mental health outcomes associated with working [32-34]. Many of the requirements for a high quality trial are being met. The results will contribute to an evidence-based approach to occupational health care for workers without an employment contract who are sick-listed due to psychological problems. Positive results in this study may lead to implementation of the Brainwork Intervention in the Netherlands. In addition, the results may offer a perspective for the further development of RTW interventions for workers sick-listed due to physical health problems.

### Methodological considerations

The design of our trial is considered feasible for the assessment of the effectiveness of a RTW intervention and fits well in the daily practice of the Dutch insurance physician. In this pragmatic trial, effectiveness instead of efficacy is studied. This increases the applicability of the intervention and has the advantage that the results will be more in line with daily occupational health practice, resulting in a high external validity [35-38].

The strength of this study is the data collection from a social security database, which contains accurate information on our primary outcome measure, duration of sick leave. Accurate information can be obtained from this database because these data are used for calculating sickness benefit claims. Register-based data, which are used for calculating earnings, are considered to be a gold standard [39-42] and prevent recall bias [40]. As a result, loss of primary outcome data due to the loss of the worker to follow-up is not expected. Thus, this study has a low risk of attrition bias. Deriving primary outcome data from the database also leads to a low risk of detection bias, despite the lack of blinding to the sick-listed workers, OH professionals and the intervention partners that are allocated to the intervention or control group. However, concerning one secondary outcome measure (number of days of paid employment during follow-up), data on RTW are also collected from self-report questionnaires because the Dutch SSA, in many cases, no longer retains data on RTW after sickness benefits have ended. As such, data collection from the database alone might underestimate RTW during the one-year follow-up.

Because the activating approach of our intervention is a well-known concept and described in detail, we expected that the results of this study are generalizable and applicable in other countries.

Results of this study will become available in 2015.
